# Number Formats, Error Mitigation, and Scope for 16‐Bit Arithmetics in Weather and Climate Modeling Analyzed With a Shallow Water Model

**DOI:** 10.1029/2020MS002246

**Published:** 2020-10-14

**Authors:** M. Klöwer, P. D. Düben, T. N. Palmer

**Affiliations:** ^1^ Atmospheric, Oceanic and Planetary Physics University of Oxford Oxford UK; ^2^ European Centre for Medium‐Range Weather Forecasts Reading UK

**Keywords:** Reduced precision, 16‐bit arithmetic, climate models, rounding error, floating‐point numbers, posit numbers

## Abstract

The need for high‐precision calculations with 64‐bit or 32‐bit floating‐point arithmetic for weather and climate models is questioned. Lower‐precision numbers can accelerate simulations and are increasingly supported by modern computing hardware. This paper investigates the potential of 16‐bit arithmetic when applied within a shallow water model that serves as a medium complexity weather or climate application. There are several 16‐bit number formats that can potentially be used (IEEE half precision, BFloat16, posits, integer, and fixed‐point). It is evident that a simple change to 16‐bit arithmetic will not be possible for complex weather and climate applications as it will degrade model results by intolerable rounding errors that cause a stalling of model dynamics or model instabilities. However, if the posit number format is used as an alternative to the standard floating‐point numbers, the model degradation can be significantly reduced. Furthermore, mitigation methods, such as rescaling, reordering, and mixed precision, are available to make model simulations resilient against a precision reduction. If mitigation methods are applied, 16‐bit floating‐point arithmetic can be used successfully within the shallow water model. The results show the potential of 16‐bit formats for at least parts of complex weather and climate models where rounding errors would be entirely masked by initial condition, model, or discretization error.

## Key Points

- Posit numbers reduce rounding errors compared to floating‐point numbers, enabling reliable shallow water simulations with 16‐bit arithmetic
- Errors caused by 16‐bit arithmetic are reduced with critical computations in 32 bit, which can be implemented on present‐day hardware
- 16‐ or even 8‐bit communication between processors introduces negligible errors, providing a perspective for reduced data communication

## 1 Introduction

Predictions of weather and climate remain very difficult despite the use of the world's fastest supercomputers. Although the available computational resources have vastly increased over the last decades, forecast errors remain and have several origins (Palmer, 2012, 2015). They can be categorized as initial and boundary condition errors, model errors, and discretization errors. For instance, uncertainties in the observational data and their assimilation contribute to the errors in the initial conditions; discrepancies between the mathematical model and the real world cause model errors; and the finite spatial and temporal resolution result in discretization errors. The forecast error is in general a combination and respective contributions can be different for different variables and forecast lead times. 64‐bit double‐precision floating‐point numbers (Float64) are used as the default option for weather and climate models since the 1980s with the rise of 64‐bit computing. The Float64 format introduces rounding errors that are largely negligible compared to the other mentioned sources of error.

Faster calculations and communication on computing architectures can be achieved with reduced precision floating‐point numbers, with a trade‐off between speed and precision. Deep learning algorithms require only low numerical precision but high computational performance. The recent boom of machine learning applications increased the demand on hardware‐accelerated reduced precision calculations, such that hardware developments increasingly offer more flexibility on low‐precision number formats. While 16‐bit arithmetic was not available for use on commodity supercomputing hardware in the past, today most hardware vendors offer the use of 16‐bit formats, such as 16‐bit half precision floating‐point numbers (Float16), on the next generation of hardware.

Graphic processing units (GPUs) started to support Float16 for increased performance (Markidis et al., 2018). Google's tensor processing units (TPUs,  Jouppi et al., 2017, 2018) support the 16‐bit BFloat16 format, a truncated version of 32‐bit single‐precision floats (Float32), as this format is sufficiently precise for many deep learning applications (Burgess et al., 2019; Gupta et al., 2015; Kalamkar et al., 2019). The world's fastest supercomputers have reached peak performances of 100 petaflop/s (10^17^ floating‐point operations per second) with Float64 in the last years, but peak performances with Float16 are already beyond the exascale milestone (10^18^ flop/s,  Kurth et al., 2018).

A gained speed from low‐precision calculations can free resources to increase the complexity and therefore the forecast skill in weather and climate models. The European Centre for Medium‐Range Weather Forecasts reduces the runtime by 40% but not the forecast skill in their forecast model when using almost entirely Float32 instead of Float64 (Váňa et al., 2017). MeteoSwiss profited similarly with Float32 in their forecast model (Rüdisühli et al., 2013). For the European ocean model NEMO, a mix of 32‐bit and 64‐bit arithmetic is a promising approach to keep accuracy‐critical parts in high precision while increasing performance in others (Tintó Prims et al., 2019).

Software emulators for other number formats than Float32 and Float64 are often used to investigate rounding errors caused by lower‐precision formats (Dawson & Düben, 2017). Although emulators are considerably slower than hardware‐accelerated formats, they allow a scientific evaluation of the introduced errors without requiring specialized hardware, such as field‐programmable gate arrays (FPGAs, Russell et al., 2017). Unfortunately, the computational performance cannot be assessed with software emulators.

Reducing the precision raises questions of the real bitwise information content. In simplistic chaotic models, only a minority of bits contain real information (Jeffress et al., 2017), providing an information theoretic argument for reduced precision calculations. Recent research covers reduced precision in floating‐point arithmetic in parts of weather forecast models, such as the dynamical core (Chantry et al., 2019; Düben et al., 2014; Hatfield et al., 2020; Thornes et al., 2017); physical parameterizations (Saffin et al., 2020); the ocean model (Tintó Prims et al., 2019); the land‐surface model (Dawson et al., 2018); and data assimilation (Hatfield et al., 2017, 2018). In contrast to those studies, we will evaluate various 16‐bit arithmetics, as other formats than floats have gained little attention, discuss options for reduced precision approaches, and present ways to mitigate rounding errors.

Although floating‐point numbers are the dominating number format in scientific computing, alternatives have been proposed (Gustafson & Yonemoto, 2017; Johnson, 2020). Posit™ numbers claim to provide more effective precision in algorithms of machine learning and linear algebra (Gustafson, 2017; Langroudi et al., 2019), compared to floats at the same word length. Posits were initially tested in simplistic weather and climate simulations (Klöwer et al., 2019)—research that is extended here—providing a more thorough investigation of various 16‐bit number formats.

Is 16‐bit arithmetic useful within weather and climate models? Which 16‐bit formats are most promising, and can model simulations be made resilient against a reduction in precision to 16 bits? These questions are covered in this study. We apply several types of 16‐bit arithmetic and test their impact on the simulated dynamics in a shallow water model that serves as a medium complexity weather and climate application.

The study is structured as follows. Section 2 outlines the different number formats, the concept of decimal precision, and mitigation methods to increase a model's tolerance for lower precision and a limited dynamic range with 16‐bit formats. Section 3 presents results of various implementations of 16‐bit arithmetic in the shallow water model. Section 4 discusses the results and provides the conclusions.

## 2 16‐Bit Number Formats and Mitigation Methods

This section discusses different types of 16‐bit arithmetic and similarities and differences between them. Mitigation methods that allow for the use of 16‐bit arithmetic within weather and climate applications are introduced.

### 2.1 16‐Bit Number Formats

#### 2.1.1 The Integer and Fixed‐Point Number Format

The simplest way to represent a real number in bits is the integer format. An *n*‐bit signed integer starts with a sign bit followed by a sequence of integer bits that are decoded as a sum of powers of 2 with exponents 0, 1, … , *n* − 2. The largest representable number *maxpos* for a signed integer format is 2^*n* − 1^ − 1. Fixed‐point numbers extend the integer format by adding *n*_*f*_ fraction bits to decode an additional sum of powers of 2 with negative exponents −1, − 2, … , − *n*_*f*_. Every additional fraction bit reduces the number of integer bits. For example, Q6.10 is the 16‐bit fixed‐point format with 6 signed integer bits and 10 fraction bits.

The arithmetic of fixed‐point numbers is very similar to integers. The range of representable numbers with integer arithmetic can therefore be changed with fixed‐point numbers, providing some flexibility for integer arithmetics (Russell et al., 2017). However, the width of the dynamic range, $\log_{10}(maxpos/minpos)$, is always limited and less than 5 orders of magnitude for any 16‐bit integer or fixed‐point format—too small for most applications. We will therefore focus on the discussion of the other number formats in the rest of the study.

#### 2.1.2 The Floating‐Point Number Format

The IEEE standard on floating‐point arithmetic defines how floats encode a real number *x* in terms of a sign and several exponent and significant bits

(1) $$x={\left(-1\right)}^{sign\;bit}\cdot 2^{e-bias}\cdot (1+f).$$

The exponent bits *e* are interpreted as unsigned integers, such that *e* − *bias* converts them effectively to signed integers. The fraction (or significant) bits *f*_*i*_ are defined as before, such that the significand (1 + *f*) is in the bounds [1, 2). An 8‐bit float encodes a real number with a sign bit (red), $n_{e}=3$ exponent bits (blue) and $n_{f}=4$ fraction bits (black) as illustrated in the following example:

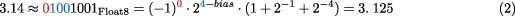

with $bias=2^{n_{e}-1}-1=3$. Exceptions to Equation 1 occur for subnormal numbers, infinity (Inf), and Not‐a‐Number (NaN) when all exponent bits are either zero (subnormals) or one (Inf when f = 0, or NaN else). 16‐bit half‐precision floating‐point numbers (Float16) have 5 exponent bits and 10 significant bits. A truncated version of the Float32 format (8 exponent bits, 23 significant bits) is BFloat16 with 8 exponent bits and 7 significant bits. A format with more exponent bits has a wider dynamic range of representable numbers but lower precision, as fewer bits are available for the significand. All floating‐point formats have a fixed number of bits in the significand; consequently, they have a constant number of significant digits throughout their range of representable numbers (subnormals excluded).

#### 2.1.3 The Posit Number Format

**Figure 1:**
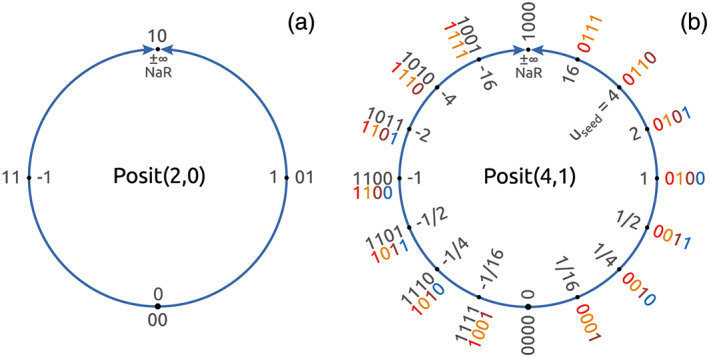
Two posit number formats obtained by projecting the real axis onto a circle. (a) 2‐bit Posit(2,0) and (b) 4‐bit Posit(4,1). The bit patterns are marked on the outside and the respective values on the inside of each circle. Bit patterns of negative numbers (black) have to be converted to their two's complement (colors) first (see text). At the top of every circle is complex infinity (±*∞*) or Not‐a‐Real (NaR). After Gustafson and Yonemoto (2017).

Posit numbers arise from a projection of the real axis onto a circle (Figure 1), with only one bit pattern for zero and one for Not‐a‐Real (NaR, also called *complex infinity*), which serves as a replacement for NaN as well as positive and negative infinity. The circle is split into *regimes*, determined by a constant *useed*, which always marks the north‐east on the posit circle (Figure 1b). Regimes are defined by *useed*^±1^, *useed*^±2^, *useed*^±3^, and so forth, which set a wide dynamic range of representable numbers. To encode these regimes into bits, posit numbers use regime bits which extend the standard on floating‐point arithmetic. Regime bits are a sequence of identical bits after the sign bit, terminated by an opposite bit. As the number of regime bits is not fixed but flexible, the significand increases in length for numbers toward ±1, when fewer regime bits are needed. Consequently, a higher precision around ±1 can be achieved with posits, which is traded against a gradually lower precision for very large or very small numbers.

A positive posit number *p* is decoded as (Chen et al., 2018; Gustafson, 2017; Gustafson & Yonemoto, 2017; Klöwer et al., 2019)

(3) $$p={\left(-1\right)}^{sign\;bit}\cdot useed^{k}\cdot 2^{e}\cdot (1+f).$$

*k* is the number of regime bits, *e* is the unsigned integer encoded by the exponent bits, and *f* is the fraction which is represented in the fraction (or significant) bits. The base of the regime bits $useed=2^{2^{e_{s}}}$ is determined by the number of exponent bits *e*_*s*_. More exponent bits $e_{s}=1,2,3,\ldots$ increase useed=4,16,256,… and therefore widen the dynamic range of representable numbers but reduce the precision around ±1. The exponent bits themselves fill gaps of powers of 2 spanned by *useed* and so do not affect the dynamic range directly by changing the value of 2^*e*^ in Equation 2. Consequently, every posit number, except for zero and NaR, can be written as $p=\pm 2^{i}\cdot (1+f)$ for a given integer *i*. In the following, a posit number format with *n* bits including *e*_*s*_ exponent bits is denoted as Posit(*n*, *e*_*s*_).

We provide an example in the Posit(8,1) system (i.e., useed=4), which encodes the number *π* as follows:

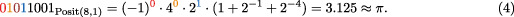

The sign bit is red, regime bits orange, the terminating regime bit brown, the exponent bit blue, and the fraction bits are black. The *k* value is derived from the number of regime bits *n*_*r*_, depending on whether those are 0 or 1: $k=-n_{r}$ if the regime bits are 0, but *k* = *n*_*r*_ − 1 if the regime bits are 1. In the example of Equation 4, k=0 for 1 regime bit of value 1. The exponent bits are interpreted as unsigned integer and the fraction bits follow the IEEE floating‐point standard for significant bits.

In order to use posits on a conventional processor, we developed for the Julia programming language (Bezanson et al., 2017) the posit emulator *SoftPosit.jl* (Klöwer & Giordano, 2019) as bindings for the C‐based library SoftPosit (Leong, 2020). A standardized posit processor is not yet available, but current research focuses on hardware implementations (Chaurasiya et al., 2018; Chen et al., 2018; Glaser et al., 2017; van Dam et al., 2019; Zhang & Ko, 2020).

### 2.2 Decimal Precision and Summary of Number Formats

Most arithmetic operations include rounding of an exact result *x*_exact_ to a representable number *x*_repr_. Based on the decimal rounding error $\left|\log_{10}\left(\frac{x_{\text{repr}}}{x_{\text{exact}}}\right)\right|$, the decimal precision is defined as (Gustafson, 2017; Klöwer et al., 2019)

(5) $$\text{decimal precision}=-\log_{10}\left|\log_{10}\left(\frac{x_{\text{repr}}}{x_{\text{exact}}}\right)\right|.$$

The decimal precision measures the number of correct decimal places after rounding. The decimal precision goes to infinity when the exact result approaches a representable number. Rounding an exact result halfway between two representable numbers maximizes the decimal error and minimizes the decimal precision. This minimum is the *worst‐case* decimal precision, which measures the number of decimal places that are at least correct after round to nearest. In the following, we will refer to the worst‐case decimal precision simply as decimal precision. The machine epsilon *ϵ*, a relative rounding error in floating‐point arithmetic, is commonly used to measure the precision of number formats. It is defined as the distance *δ* between 1 and the next largest representable number and can be given in terms of decimal precision as $\epsilon =-\log_{10}\left(\log_{10}\left(1+\frac{\delta }{2}\right)\right)$.

**Figure 2:**
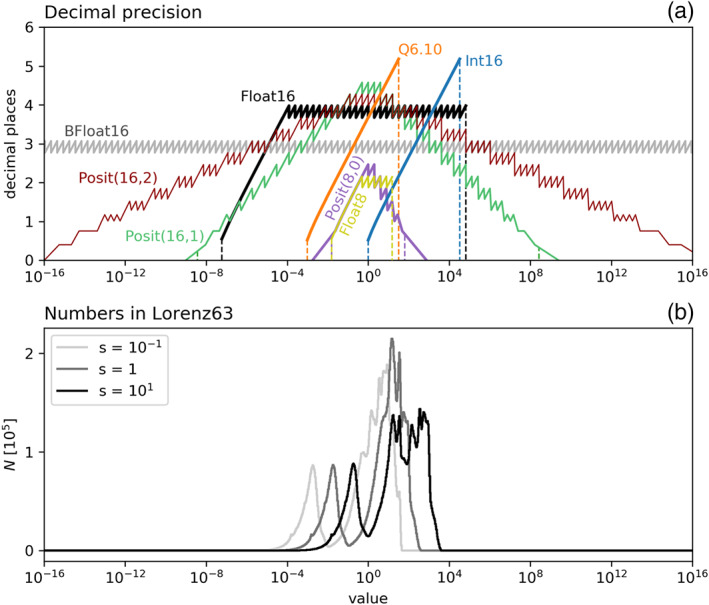
(a) Decimal precision for various number formats. Dashed vertical lines indicate for each format the range from *minpos* to *maxpos* of representable numbers. Float64, Float32, and Posit32 are beyond the axes limits. (b) Histogram of results of the absolute values of all arithmetic operations in Lorenz system rescaled by *s*.

The decimal precision for various 16‐ and 8‐bit floats and posits, 16‐bit integers, and the fixed‐point format Q6.10 (6 integer bits, 10 fraction bits) is presented in Figure 2a. Floats have an exponent that is evenly spaced in logarithmic space, which results in a nearly constant decimal precision. The deviations from a constant decimal precision, which can be seen as regular spikes in Figure 2a, are due to a linearly spaced significand. The decimal precision for floats decreases for the subnormal numbers toward the smallest representable number *minpos*. Posits have an increased decimal precision around 1 and a wide dynamic range, due to the tapered precision toward *minpos* and the largest representable number *maxpos*. The decimal precision for posits is above zero outside the dynamic range as posits have a no overflow/no underflow rounding mode. Integers are linearly spaced, and consequently, their precision increases toward *maxpos* for 16‐bit signed integers. The decimal precision of fixed‐point numbers has an identical shape but is shifted toward smaller numbers by a factor of $\frac{1}{2}$  for each additional fraction bit. Consequently, many arithmetic calculations should be placed close to *maxpos*, but the small range of high precision is restrictive for many applications. No convincing results with integer or fixed‐point arithmetic were achieved in this study, especially as the limited dynamic range of less than 5 orders of magnitude imposes a very difficult constrain to avoid underflows or overflows.

Characteristics of various formats are summarized in Table 1. Float64 has more than 10^15^ bit patterns reserved for NaN, but these only make up <0.05% of all available bit patterns. However, the percentage of redundant bit patterns for NaN increases for floats with fewer exponent bits and poses a noticeable issue for Float16 and Float8. Every posit number format has only one bit pattern reserved for NaR, such that this issue is negligible for posits.

**Table 1:** Characteristics of Various Number Formats

Format	Bits	Exp bits	*minpos*	*maxpos*	*ϵ*	% NaR
Float64	64	11	5.0 · 10^−324^	1.8 · 10^308^	16.3	0.0
Float32	32	8	1.0 · 10^−45^	3.4 · 10^38^	7.6	0.4
Float16	16	5	6.0 · 10^−8^	65,504	3.7	3.1
BFloat16	16	8	9.2 · 10^−41^	3.4 · 10^38^	2.8	0.4
Float8	8	3	1.5 · 10^−2^	15.5	1.9	12.5
Posit32	32	2	7.5 · 10^−37^	7.5 · 10^37^	8.8	0.0
Posit(16,1)	16	1	3.7 · 10^−9^	3.7 · 10^9^	4.3	0.0
Posit(16,2)	16	2	1.4 · 10^−17^	1.4 · 10^17^	4.0	0.0
Posit(8,0)	8	0	1.5 · 10^−2^	64	2.2	0.4
Int16	16	0	1	32,767	0.8	0
Q6.10	16	0	9.8 · 10^−4^	32.0	3.7	0

*Note*. *minpos* is the smallest representable positive number and *maxpos* the largest. The machine error *ϵ* is here given as decimal precision (i.e., correct decimal places after rounding). % NaR denotes the percentage of bit patterns that represent Not‐a‐Number (NaN), infinity, or Not‐a‐Real (NaR).

### 2.3 Approaches and Mitigation Measures to Allow for the Use of 16‐Bit Number Formats in Weather and Climate Applications

#### 2.3.1 Mixed Precision Arithmetic

In many models, it will not be possible (or useful) to use 16‐bit arithmetic throughout the entire model. Some model components will be more sensitive to a reduction in precision when compared to others, and it often makes sense to reduce precision only in those components where results are not deteriorated, while keeping precision high in precision‐sensitive components. This approach is called *mixed precision* and is already used for the reduction to single precision in ocean and atmosphere models (Váňa et al., 2017; Tintó Prims et al., 2019).

#### 2.3.2 Algorithmic Changes: Rescaling, Reordering, and Precomputations

Equations can be *rescaled* via multiplication with a constant rescaling factor *s* to shift the dynamic range occurring in an algorithm toward larger numbers (for *s* > 1) or toward smaller numbers (*s* < 1). Rescaling can be used to adjust the number range to the decimal precision of the number formats. If nonlinear terms are considered, this multiplicative rescaling is ineffective, as the rescaling factor *s* appears inside the nonlinear terms. The nonlinear terms are therefore effectively invariant under multiplicative rescaling and only the linear terms are scaled by *s*. Figure 2 includes histograms of numbers occurring in the rescaled Lorenz system (Jeffress et al., 2017; Kwasniok, 2014; Lorenz, 1963; Tantet et al., 2018) as an example from Klöwer et al. (2019). For posit arithmetic, it is preferable to use $s=\frac{1}{10}$ in the Lorenz system to scale the prognostic variables to match the range of highest decimal precision around ±1, which increases the complexity of the Lorenz attractor and decreases the average rounding error.

Furthermore, it is sometimes possible to avoid intermediate arithmetic results, which may be outside the dynamic range of a number format, by changing the order in which multiplications and divisions are executed. In general, it is preferable to combine such operations to a single multiplication with a constant which can be precomputed. Although this will have a negligible effect on the rounding error for floating‐point arithmetic due to the approximately constant decimal precision throughout the range of numbers (subnormals excluded), it reduces the risk of overflow or underflow. Reordering the shallow water equations is discussed in section 3.1.

#### 2.3.3 Reduced Precision Communication

Complex weather and climate models rely on parallelization to distribute the computational cost of simulations efficiently among the processing units in a large cluster or supercomputer. Parallel execution typically requires domain decomposition, where the spatial domain is split into many subdomains to be calculated separately on individual processing units. Domain decomposition requires communication of the boundary values of a subdomain with the neighboring subdomains. If 16‐bit arithmetic cannot be used within the entire model, it may still be possible to reduce precision in the communication between processors.

Not all weather and climate models would benefit from a reduced precision communication as the acceleration potential depends on many factors specific to a model and the used hardware (e.g., number of nodes in a cluster and how shared and distributed memory is managed). It will also be important whether communication is latency or volume bound. Latency bound communication is bound by the time a package of information requires to travel between processors. In contrast, volume bound communication is limited by the bandwidth that is available for communication. Only the latter will benefit from a reduction in data volume, which can be achieved with reducing precision.

However, if communication volume is an identified bottleneck in a given application, which is often the case in weather and climate models, reliable model simulations might be possible with 16‐ or even 8‐bit communication, allowing for a significant reduction in computing time. In general, various lossy and lossless data compression techniques can be used to reduce communication volume (Fan et al., 2019). Lossless communication allows for bit‐reproducible results when compared to simulations that do not use compressed communication. We therefore restrict ourselves to lossy‐type conversions which introduce rounding errors to the data that are sent around while the overhead due to encoding before and decoding after communication remains small.

## 3 A Shallow Water Model With 16‐Bit Arithmetic

In this section, the different number formats Float16, BFloat16, Posit(16,1), and Posit(16,2) are evaluated when solving the shallow water equations. The shallow water model used here is an updated version of the one used in Klöwer et al. (2019), and most details described therein are also valid here. A vertical integration of the Navier‐Stokes equations yields the shallow water equations that can be used to understand many features of the general circulation of atmosphere and ocean as well as some two‐dimensional nonlinear interactions on shorter length and time scales (Gill, 1982; Vallis, 2006). The shallow water equations for the prognostic variables velocity u=(u,v) and sea surface elevation *η* are

(6a) $$\frac{\partial u}{\partial t}+(u\cdot \nabla )u+f\hat{z}\times u=-g\nabla \eta +D+F$$

(6b) $$\frac{\partial \eta }{\partial t}+\nabla \cdot (uh)=0.$$

*η* can be interpreted as pressure for the atmosphere (Gill, 1982). The shallow water system is forced with a zonal wind stress **F**. The dissipation term **D** removes energy on large scales (bottom friction) and on small scales (diffusion). The nonlinear term (**u** · ∇)**u** represents advection of momentum. The term $f\hat{z}\times u$ is the Coriolis force, and −*g* ∇ *η* is the pressure gradient force, with *g* being the gravitational acceleration. Equation 6b is the shallow water variant of the continuity equation, ensuring conservation of mass.

The shallow water equations are solved in the (*x*, *y*) plane over the zonally periodic rectangular domain *L*_*x*_ × *L*_*y*_, of size 2,000 × 1,000 km. We associate *x* with the zonal and *y* with the meridional direction. The domain is centered at 45°N with the beta‐plane approximation (Vallis, 2006). The boundary conditions are periodic in zonal direction and no slip at the northern and southern boundaries. The layer thickness is h=η+H(x), with

(7) $$H(x)=H_{0}-H_{1}\exp\left(-H_{\sigma }^{-2}{\left(x-\frac{L_{x}}{2}\right)}^{2}\right)$$

being the undisturbed depth, representing a meridional mountain ridge at $x=\frac{L_{x}}{2}$ spanning from the southern to the northern boundaries. The standard depth is $H_{0}=500$ m. The ridge has a maximum height of $H_{1}=50$ m and a characteristic width of $H_{\sigma }=300$ km, which makes the zonal current barotropically unstable. The flow regime is therefore governed both by eddy‐mean flow as well as eddy‐eddy interactions.

The time step Δt=282 s is chosen to resolve surface gravity waves, traveling at an estimated phase speed of $\sqrt{gH_{0}}$ with a Courant‐Friedrichs‐Lewy (CFL) number close to 1 and gravitational acceleration g=10 ms^−1^. The wind stress forcing $F=(F_{x},0)$ is constant in time, acts only on the zonal momentum budget

(8) $$Fx=\frac{F_{0}}{\rho h}\cos{\left(\pi \left(y{L_{y}}^{-1}-1\right)\right)}^{2},$$

and vanishes at the boundaries. The water density is ρ=1,000 kg m^−3^ and $F_{0}=0.12$ Pa. The wind forcing acts as a continuous input of large‐scale kinetic energy that is balanced by the dissipation term.

The dissipation term **D** is the sum

(9) $$D=-\frac{c_{D}}{h}\|u\|u-\nu \nabla^{4}u$$

of a quadratic bottom drag with dimensionless coefficient $c_{D}=10^{-5}$ (Arbic & Scott, 2008) and a biharmonic diffusion with viscosity coefficient *ν* ≈ 1.33 × 10^11^ m^4^s^−1^ (Griffies & Hallberg, 2000).

The shallow water equations are discretized using second‐order centered finite differences on an Arakawa C‐grid (Arakawa & Lamb, 1977) and the fourth‐order Runge‐Kutta method (Butcher, 2016) is used for time integration of the pressure, Coriolis, and advective terms, whereas a semi‐implicit method is used for the dissipative terms **D**. We present results of simulations with three different levels of resolution: high‐resolution simulations with a grid spacing of Δ=5 km (400x200 grid points), medium resolution simulations with a grid‐spacing of Δ=20 km (100 × 50 grid points) and low resolution with a grid spacing of Δ=40 km (50 × 25 grid points). The advection terms are discretized using an energy and enstrophy conserving scheme (Arakawa & Hsu, 1990).

The shallow water equations are extended with an advection equation for tracers. Temperature and humidity or salinity are examples of tracers in the atmosphere and the ocean. For simplicity, we regard them as passive here, such that they do not influence the flow. The advection of a passive tracer *q* given a velocity **u** is governed by

(10) $$\frac{\partial q}{\partial t}+u\cdot \nabla q=0.$$

A semi‐Lagrangian advection scheme (Smolarkiewicz & Pudykiewicz, 1992) is used to discretize Equation 10. In this discretization, the tracer concentration for a given grid cell (i.e., the arrival point) is calculated from the concentration at a departure point at a previous time step. The departure point is determined by back tracing the velocities from the arrival point. Departure points in general do not coincide with grid nodes, such that an interpolation from the surrounding grid points is required to find the concentration at the departure point, which is then defined to be the concentration at the arrival point.

### 3.1 Rescaling the Shallow Water Equations

The dynamic range of representable numbers with 16‐bit arithmetic is for most formats discussed here considerably smaller than with Float32 or 64 (Figure 2a and Table 1). It is therefore important to rescale the calculations to limit the range of arithmetic results to stay within the bounds of a given 16‐bit format.

The prognostic variables in the shallow water equations are typically $O(1{\text{ms}}^{-1})$ for **u**, O(1m) for *η*, and O(1) for *q*. Their physical units are therefore retained in the discretized numerical model, and we do not apply a rescaling of the shallow water equations as a whole. However, the grid spacing Δ in units of meter is large for geophysical flows. We therefore use dimensionless Nabla operators $\tilde{\nabla }=\Delta \nabla$. The continuity equation (Equation 6b), for example, reads then as

(11) $$\eta^{n+1}=\eta^{n}+\tilde{\Delta t}\left(-\tilde{\nabla }\cdot {\left(uh\right)}^{n}\right)$$

for an explicit time stepping scheme. $\tilde{\Delta t}$ is the rescaled time step, a Runge‐Kutta coefficient times $\frac{\Delta t}{\Delta }$ to combine a division by a large value for Δ and a subsequent multiplication with a large value for Δ*t* into a single multiplication with $\tilde{\Delta t}$. The other terms are rescaled accordingly ( $\tilde{f}=f\Delta$; $\tilde{F}=F\Delta$). As these terms remain constant, they can be precomputed at higher precision during model initialization. The momentum equations are rescaled similarly.

Diffusion is an example of a discretization scheme that requires rescaling for the arithmetic results to fit into the limited dynamic range. Biharmonic diffusion (Griffies & Hallberg, 2000) calculates a fourth derivative in space, which is often very small $O(10^{-20})$ in geophysical applications, due to the large physical dimensions, when using meters as a unit for length. Contrarily, biharmonic viscosity coefficients are typically very large $O(10^{11})$. We therefore rescale **D** accordingly

(12) $$\tilde{D}=-\frac{\tilde{c_{D}}}{h}\|u\|u-\tilde{\nu }{\tilde{\nabla }}^{4}u$$

with $\tilde{c_{D}}=c_{D}\Delta =0.2m$ and $\tilde{\nu }=\nu \Delta^{-3}\approx 0.16\;{\text{ms}}^{-1}$, which are precomputed. The term $\tilde{D}$ is computed instead of **D** and the scaling eventually undone when multiplying with the rescaled time step $\tilde{\Delta t}$.

The semi‐Lagrangian advection scheme is reformulated for 16‐bit arithmetics. In the absence of sources and sinks, the Lagrangian point of view states that the tracer concentration *q* does not change following a flow trajectory. The concentration *q* at departure points **x**_*d*_ at time *t* is therefore the same as the concentration at time *t* + Δ*t*_adv_ at arrival points **x**_*a*_, which are chosen to coincide with the grid points. Based on the flow velocity at the arrival point, the departure point is derived. To avoid large numbers for the coordinates (in our case $L_{x}=2\cdot 10^{6}$ m), nondimensional departure points ${\tilde{x}}_{d,rel}$ relative to the arrival point are computed as

(13) $${\tilde{x}}_{d,rel}=-u(x_{a},t+\Delta t_{\text{adv}})\left(\frac{\Delta t_{\text{adv}}}{\Delta }\right).$$

A scaling with the grid spacing inverse Δ^−1^ is applied such that all terms are O(1) and therefore representable with 16‐bit arithmetics. In practice, when converting the relative departure point ${\tilde{x}}_{d,rel}$ to an array index for the interpolation, the floor function is used in combination with integer arithmetics. This essentially separates a computation with real numbers into two parts: one that can be computed with integers without rounding errors and a calculation with floating‐point numbers, with a removed offset to reduce rounding errors.

### 3.2 Shallow Water Simulations in 16‐Bit Arithmetic

**Figure 3:**
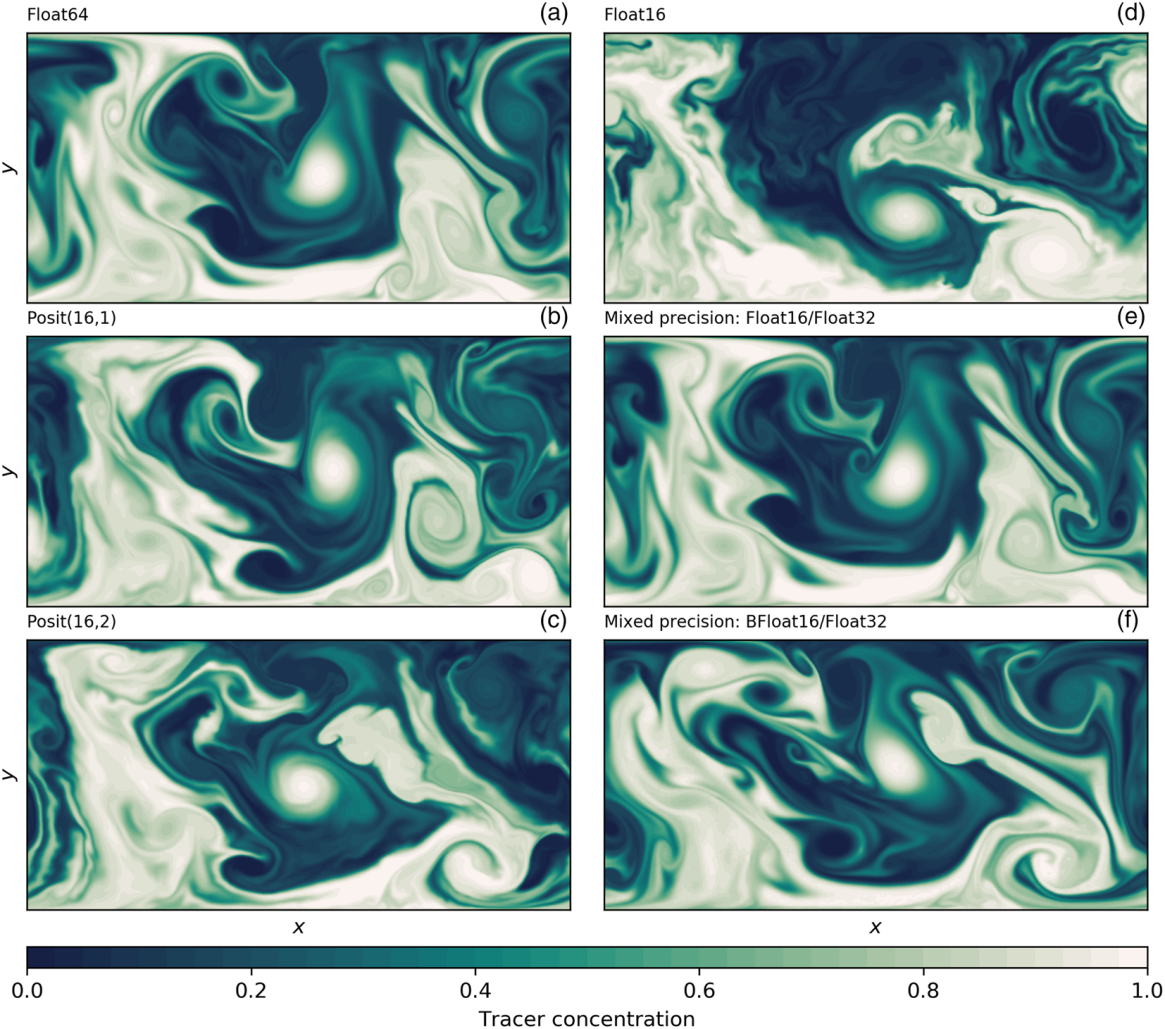
Snapshot of tracer concentration simulated by the shallow water model using different 16‐bit number formats and the high‐resolution configuration ( Δ=5 km). The mixed‐precision simulations in (e) and (f) are using Float32 for the representation of prognostic variables only. The tracer was injected uniformly in the lower half of the domain, 50 simulation days before the time step shown.

The shallow water model simulates vigorous turbulence interacting with a zonal current (Figure 3). Both float and posit arithmetic present very similar fluid dynamics in comparison to the Float64 reference in only 16 bit. A snapshot of tracer concentration many simulated days after initialization reveals turbulent mixing of the tracer that is well simulated with posits. However, with Float16, the simulation deviates faster from the reference than with Posit(16,1) and to a lesser degree with Posit(16,2), presumably due to the small‐scale instabilities visible in the snapshot as wavy filaments and fronts. These instabilities are clearly triggered by Float16 arithmetics, but to a lower degree also visible for posits. This provides some visual evidence that accumulated rounding errors are reduced with posits, especially with Posit(16,1). BFloat16 arithmetic is not able to simulate the shallow water dynamics, as tendencies are too small to be added to the prognostic variables. A stalling of the simulated flow is observed. The results with mixed precision, Float16/Float32 and BFloat16/Float32, will be discussed in section 3.3.

Short‐term forecasts at medium‐resolution ( Δ=20 km) are performed to analyze the differences between different 16‐bit arithmetics. To quantify the error growth caused by rounding errors with different arithmetics in a statistically robust way, we create a number of forecasts with each member starting from one of 200 randomly picked start dates from a 50‐year long control simulation. The forecast error in the shallow water model is computed as root mean square error (RMSE) of sea surface height *η* with respect to Float64 simulations. Other variables yield similar results. Each forecast is performed several times from identical initial conditions but with the various number formats. The error growth caused by rounding errors is additionally compared to the error introduced by discretization. A low‐resolution model configuration with Δ=40 km is used to quantify a realistic level of discretization error. The RMSE is normalized by the climatological mean forecast error at very long lead times, which is the same for all model configurations. When the normalized RMSE reaches 1, all information on the initial conditions is removed by the chaotic evolution of the shallow water system.

**Figure 4:**
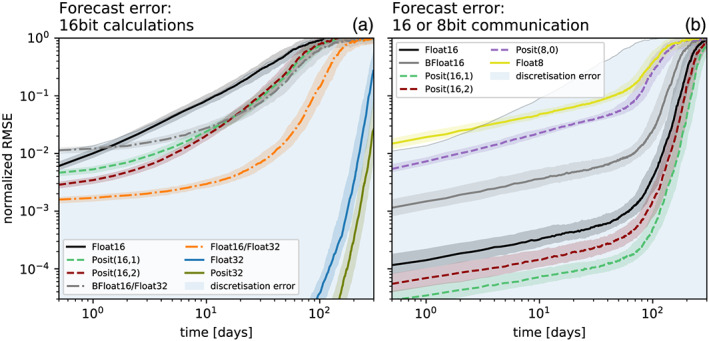
Forecast error of sea surface height *η* measured as root mean square error (RMSE) taking Float64 as reference. (a) Forecast error for various 16‐bit number formats and mixed 16‐/32‐bit simulations for which the prognostic variables are kept in Float32. (b) Forecast error for reduced precision communication in 8 or 16 bit with various number formats used for encoding, with Float64 used for all calculations. The communication of boundary values occurs at every time step for the prognostic variables. The RMSE is normalized by a mean forecast error at very long lead times. Solid lines represent the median of 200 forecasts per number format. The shaded areas of each model configuration denote the interquartile range of the forecast experiments.

The forecast error of Float16 is as large as the discretization error and clearly outperformed by 16‐bit posit arithmetic (Figure 4a). Both Posit(16,1) and Posit(16,2) yield a forecast error that is several times smaller than Float16. The forecast error of 32‐bit arithmetic is several orders of magnitude smaller and is only after 200 days as large as the error for 16‐bit arithmetic at short lead times of about 10 days. Also at 32 bit, posits clearly outperform floats.

To investigate the effect of rounding errors on the climatological mean state of the shallow water system, we zonally average the zonal velocity *u*. This average is based on 300‐day long simulations starting from 200 different initial conditions, which cover the various states in the long‐term variability of the shallow water system. However, the climatology from a single very long simulation has not been assessed.

**Figure 5:**
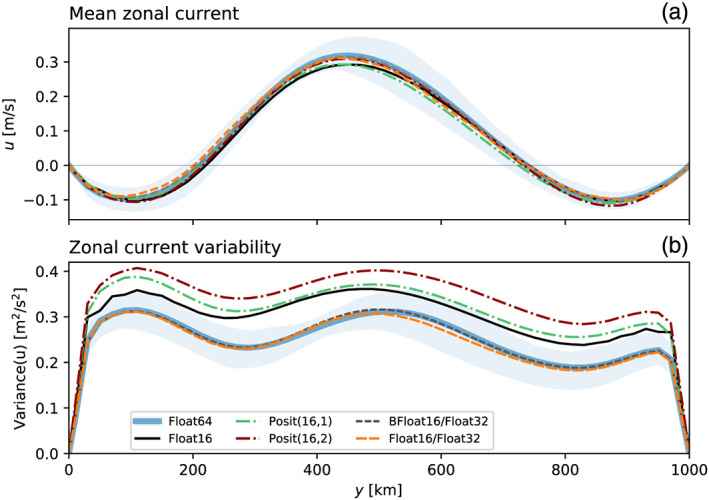
Climatology and variability of the zonal current in the medium‐resolution simulations. (a) Zonally averaged zonal current *u* as a function of the meridional coordinate *y*. (b) Zonal variance of the zonal current as a function of *y*. The dashed lines for BFloat16/Float32 and Float16/Float32 are almost identical. The shaded area denotes the interquartile temporal variability around the (a) mean and (b) variance of reference simulation with Float64.

The mean state is an eastward flow of about 0.3 m s^−1^, about 3 to 4 times weaker than individual velocities throughout the domain (Figure 5a), which is typical for turbulent flows. A weak westward mean flow is found at the northern and southern boundaries. No 16‐bit format was found to have a significant impact on the mean state. The variability of the flow around its mean state is high throughout the domain (Figure 5b). The variability is significantly increased by 10–30% with 16‐bit arithmetic, especially with Posit(16,2). This is probably caused by rounding errors that are triggering local perturbations which increase variability.

The turbulence in shallow water simulations is largely geostrophic, such that the pressure gradient force opposes the Coriolis force. The resulting geostrophic velocities **u**_*g*_ can be derived from the sea surface height *η* as

(14a) $$u_{g}=\frac{g}{f}\hat{z}\times \nabla \eta$$

(14b) $$u=u_{g}+u_{ag}$$

and deviations from the actual flow **u** are the ageostrophic velocity components **u**_*ag*_. We project both components on the actual velocities to obtain the flow‐parallel components ${ũ}_{g}$ and ${ũ}_{ag}$ via

(15) $${ũ}_{g}=\frac{u_{g}\cdot u}{\left\|u\right\|},\;{ũ}_{ag}=\frac{u_{ag}\cdot u}{\left\|u\right\|}.$$

**Figure 6:**
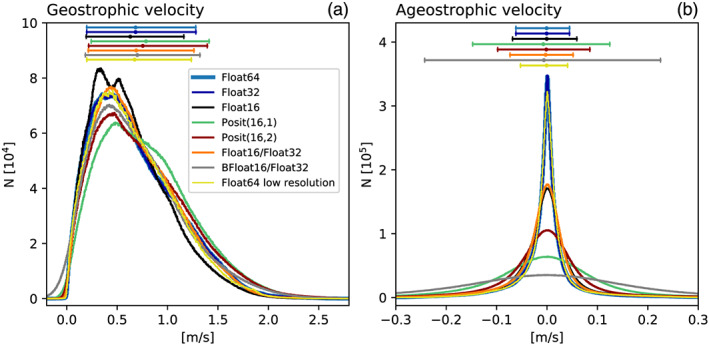
Geostrophic balance as simulated with different number formats. (a) Histograms of flow‐parallel components of geostrophic velocity. (b) as (a) but for the ageostrophic velocities. Horizontal bars denote the mean and 10th and 90th percentile in respective colors.

The geostrophic velocities in the shallow water simulations can reach up to 2 m s^−1^, are hardly negative (i.e., against the flow), and have a mean of about 0.7 m s^−1^ (Figure 6a). This behavior is well simulated with 16‐bit number formats, although posits increase the strength of geostrophic velocities slightly. Ageostrophic velocity components are found to be isotropic and are oriented equally frequent with and against the prevailing flow. They rarely exceed ±0.1  m s^−1^ and are therefore comparably small, which is expected in geostrophically balanced turbulence. Ageostrophic velocities can be seen as a measure of the physical instabilities in the flow field and their variance is indeed increased when simulated with 16‐bit number formats. Float16 and posits show clearly fewer ageostrophic velocities around 0, pointing toward an increased number of simulated instabilities. In particular, Posit(16,1) increases the variance of ageostrophic velocities by more than a factor of 2. It is unclear where in the model integration rounding errors of 16‐bit arithmetic trigger instabilities that lead to the observed increase in ageostrophy. We conclude that although the geostrophic balance in the simulations is maintained, rounding errors lead, likely due to an increase in ageostrophy, to a higher variability in the flow field.

**Figure 7:**
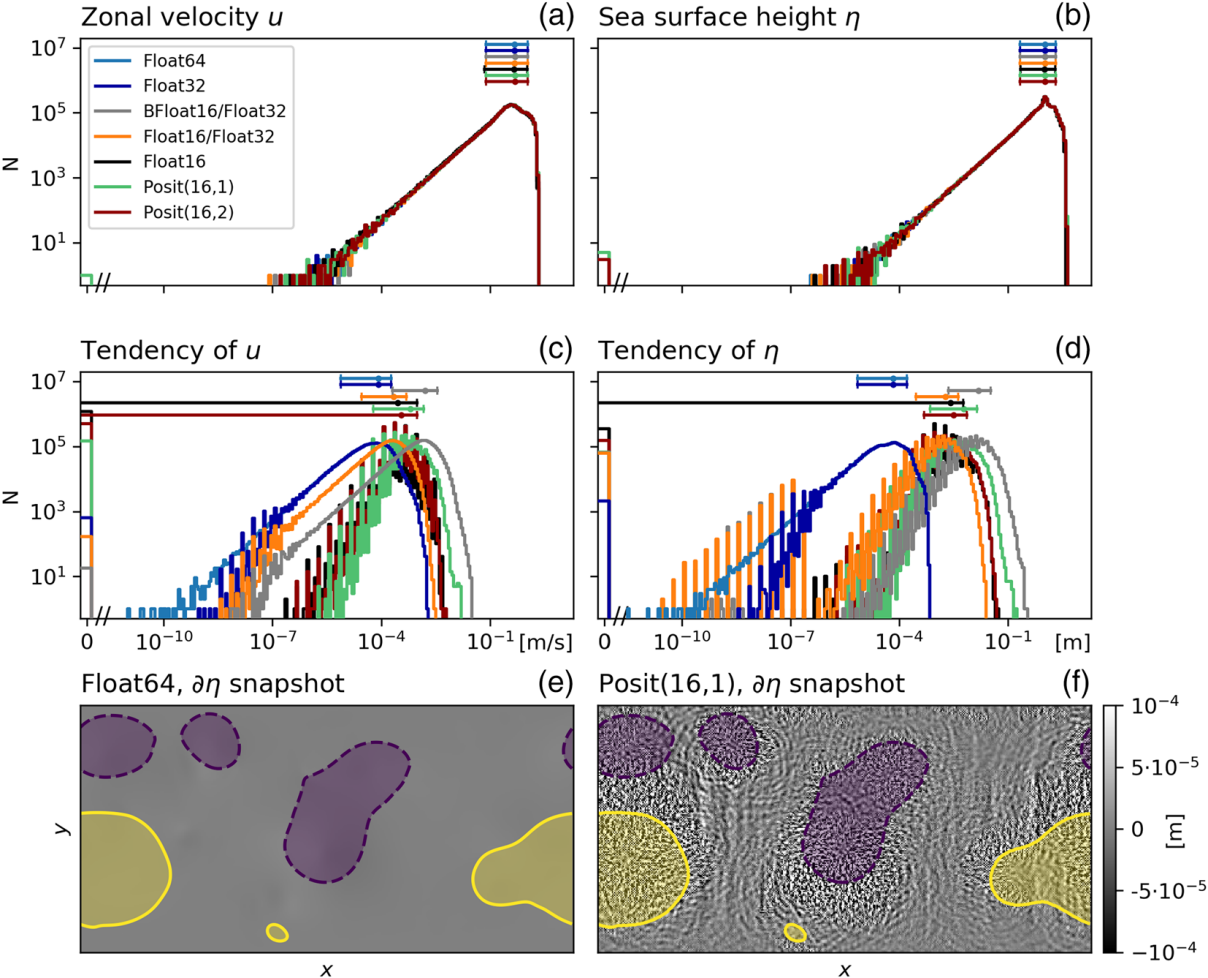
Histograms of the numeric values of the prognostic variables (a) zonal velocity *u*, (b) sea surface height *η*, and the respective tendencies of (c) *u* and (d) *η*, simulated with different 16‐, 32‐, and 64‐bit number formats. Mean and 10th and 90th percentile are shown above the histograms in respective colors. Snapshots of the tendencies of *η* simulated with (e) Float64 and (f) Posit(16,1). Snapshots are similar for other 16‐bit formats (not shown here). Areas of sea surface height anomalies exceeding ±1.4 m are shown in purple (negative) and yellow (positive). Note the break on the *x* axis close to zero in (a), (b), (c), and (d).

As 16‐bit arithmetics have no significant impact on the climatological mean state, histograms of prognostic variables are also not changed (Figures 7a and 7b). However, the tendencies are increased by orders of magnitude with 16‐bit arithmetics (Figures 7d and 7e), as rounding errors cause gravity waves to radiate away from eddies (Figure 7f). Gravity waves are identified from the tendency of sea surface height. Comparing their propagation to the location of anomalous sea surface height, which is used as a proxy to locate eddies, we assume that rounding errors in regions of high eddy activity lead to instabilities that propagate away in the form of gravity waves. These gravity waves are not present in Float64 simulations (Figure 7e) and tend to have only a small impact on quasi‐geostrophic dynamics, as they act on different time and length scales. It is unclear but possible that gravity waves cause the observed increased ageostrophic velocities for 16‐bit arithmetic.

Tendencies are about 4 orders of magnitude smaller than the prognostic variables. This poses a problem for number formats with a machine epsilon, measured as decimal precision, significantly lower than 4 decimal places (Table 1). Float16 has a machine epsilon of 3.7, which is presumably close to the lower limit beyond which the addition of tendencies will be round back. The BFloat16 number format has a machine epsilon of 2.8, which explains why the flow is stalling when simulated with BFloat16.

### 3.3 Mixed Precision Arithmetic in the Shallow Water Model

In the previous simulations, the entire shallow water simulation was performed with the specified number format. As the addition of tendencies to the prognostic variables was identified as a key calculation that is error prone, we investigate now the benefits of mixed precision arithmetic, where Float32 is used for the prognostic variables, but the tendencies are computed with either Float16 or BFloat16, two number formats that have the lowest decimal precision around 1. The prognostic variables are now reduced to Float16 or BFloat16 before calculations of the right‐hand side and every term of the tendencies is converted back before addition to the prognostic variables. Subscripts 16 and 32 were used to denote variables held at 16‐ and 32‐bit precision, respectively, and we let Float32() be the conversion function. The continuity equation (Equation 6b) then becomes

(16) $$\frac{\partial \eta_{32}}{\partial t}=-\text{Float32}(\partial_{x}(u_{16}h_{16})\;+\;\partial_{y}(v_{16}h_{16}))$$

and similar for *u* and *v* in Equation 6a.

Snapshots of tracer concentration reveal well‐simulated geostrophic turbulence (Figures 3e and 3f) with Float16/Float32 or BFloat16/Float32, and instabilities at fronts or in filaments are visibly reduced compared to pure 16‐bit arithmetic. The forecast error is strongly reduced once the prognostic variables are kept as Float32 (Figure 4a), supporting the hypothesis that the addition of tendencies to the prognostic variables is a key computation with low rounding error tolerance. Despite BFloat16 not being suitable for shallow water simulations when applied to all computations, mixing BFloat16 with Float32 arithmetic yields a similar error growth to posits, which is well below the discretization error. Mean state or variability are virtually identical for both mixed precision cases (Figure 5) compared to the Float64 reference. The geostrophic balance is largely unaffected, but ageostrophic velocities increase in variance, especially for BFloat16 (Figure 6). Gravity waves are similarly present for mixed precision although weaker for tendencies computed with Float16 (Figure 7d), and as discussed, they tend to not interact with the geostrophic time and length scales. Although the results show that Float16 is generally a preferable number format over BFloat16 for the applications presented here, we acknowledge that the conversion between Float32 and Float16 will come with some computational cost. In contrast, the conversion between BFloat16 and Float32 is computationally very cheap as both formats have the same number of exponent bits. Removing significant bits, applying rounding, and padding trailing zeros are the only operations for this conversion. Following the results here, mixing 16‐ and 32‐bit precision is found to be an attractive solution to circumvent spurious behavior due to 16‐bit floating‐point arithmetics. Performance benefits are still possible as most calculations are performed with 16 bit, with error‐critical computations in 32 bit to reduce the overall error.

Using mixed precision in our shallow water model, 77% of the arithmetic operations are performed in 16 bit and the remaining 23% in 32 bit. Assuming Float16/BFloat16 to be 2 times faster than Float32 and conversion costs to be negligible, this would yield another 40% reduction in computing time on top of a reduction from Float64 to Float32. However, this depends on the soft and hardware implementation considered. Some of the 16‐bit accelerators (GPU/TPU) can increase the flop rate by more than a factor of 2 when compared to Float32. In addition, the shallow water model regarded here has a comparably simple right‐hand side, such that more complex models will spend more time to compute tendencies which will come with a larger performance increase.

Mixed precision is an attractive solution as hardware‐accelerated 16‐bit floating‐point arithmetic is already available on GPU or TPU and implementations therefore do not rely on the development of future computing hardware, which is the case for posits.

### 3.4 Reduced Precision Communication for the Shallow Water Model

A standard method to parallelize simulations is the distributed‐memory parallelism via Message Passing Interface (MPI). We emulate MPI‐like communication in the shallow water model with the copying of boundary values between the right and left boundaries (periodic boundary conditions). Although the shallow water model does not run in parallel, reducing the precision in the copying of boundary values introduces an equivalent error as if reduced precision MPI communication was used between subdomains. Reduced precision is applied for the communication of the prognostic variables at every Runge‐Kutta substep.

**Figure 8:**
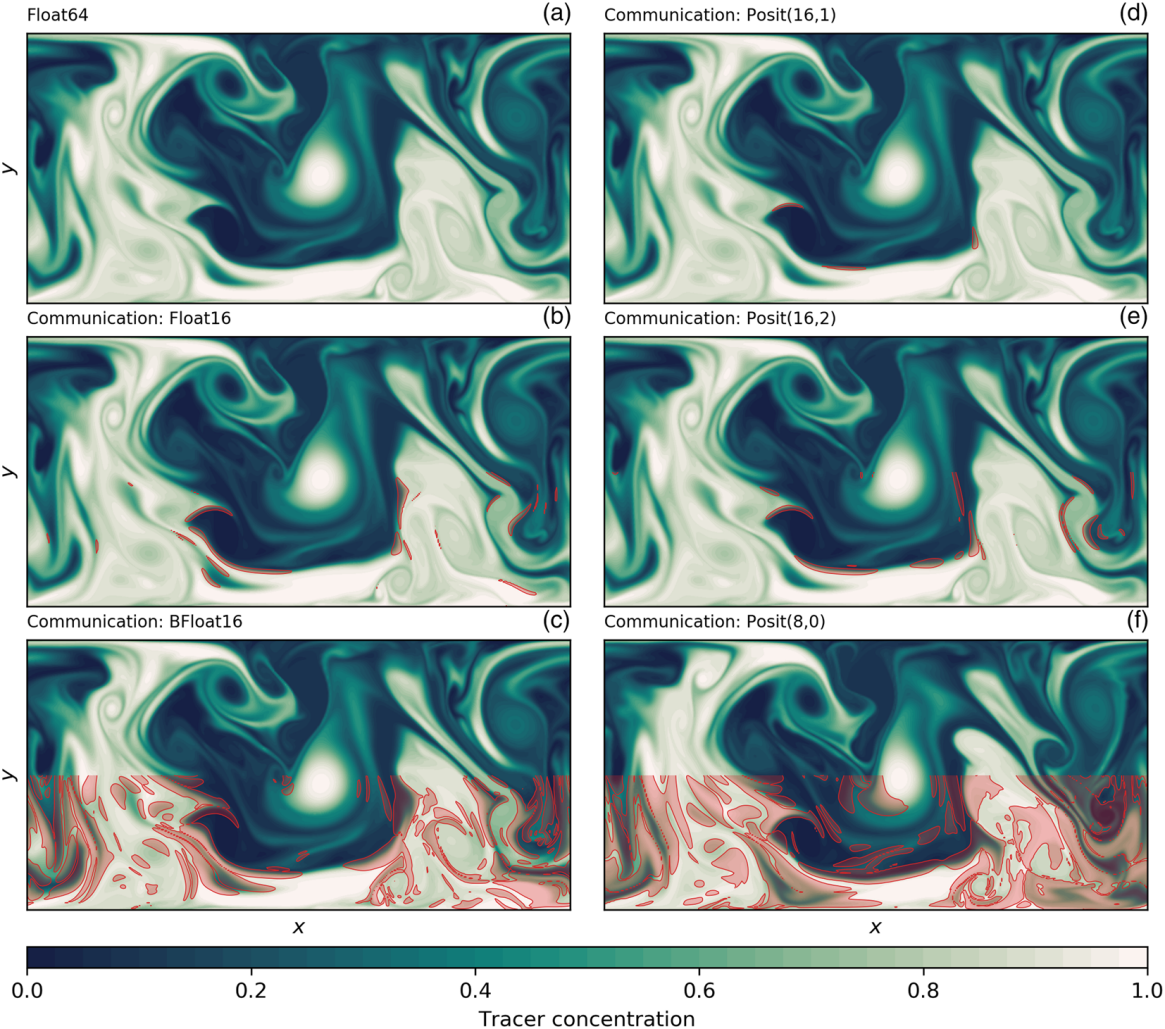
Snapshot of tracer concentration simulated by the shallow water model using reduced precision communication. The communication of boundary values occurs at every time step for the prognostic variables. Float64 was used for all calculations. Areas where the absolute error exceeds 0.05 are shaded in red only in the lower half of the domain. The tracer was injected uniformly in the lower half of the domain 50 days before. This simulation was run in the high‐resolution configuration ( Δ=5 km).

Snapshots of tracer concentration simulated with reduced precision communication show a negligible error for Float16 and posits (Figure 8). The error is largest at fronts and not concentrated around the boundaries. Encoding the communication with BFloat16 introduces a larger error than for the other 16‐bit formats as the decimal precision is with 2.8 clearly lower (Table 1) for the range of values occurring within the prognostic variables (Figures 7a and 7b). The errors are quantified by the RMSE of surface height *η* as before and are up to about 2 orders of magnitude smaller than the errors that result from 16‐bit arithmetic. As even the worst 16‐bit communication format, BFloat16, has a smaller error than the best mixed precision formats, Float16 with Float32, we extend the short‐term forecast experiments to include two 8‐bit formats, Posit(8,0) and Float8 (see Table 1 for a description). Both formats are found to be suitable for reduced precision communication here and do not introduce an error that is larger than the discretization error. Having said that, Float8 communication introduces an error that is comparably large in the first days but growths only linearly in the first 50 days of the simulation, which is in contrast to the exponential error growth observed for 16‐bit arithmetic.

## 4 Conclusion and Discussion

Future high performance computing architecture will support some 16‐bit arithmetics. The wall clock time for weather and climate simulations could be greatly reduced if computationally demanding algorithms were run at such reduced precision. We tested a number of options for 16‐bit arithmetic for weather and climate applications in a shallow water model. The best results were achieved with 16‐bit posits (with either 1 or 2 exponent bits) which appear very promising for application in high performance computing for Earth system modeling. Float16 can be used to perform forecasts with the shallow water model while the application of BFloat16 or integer arithmetic was not successful.

In general, 16‐bit arithmetics were not found to alter the climatological mean state or the large‐scale dynamics. However, variability and ageostrophic velocities were increased, such that second‐ and higher‐order statistics should undergo testing to assess the model reliability. Depending on the application, an increased variability does not necessarily deteriorate the model, especially for more realistic model setups than considered here. However, our findings suggest that reduced precision changes need to be done carefully as specific simulation features can change without obvious impact on mean diagnostics.

Shallow water simulations with 16‐bit arithmetic required rescaling of some terms but no major revisions of the model code or algorithms. Given that only floats are currently hardware supported, we investigated mixed precision approaches. Keeping the prognostic variables at 32 bit while computing the tendencies in 16 bit reduced the rounding errors significantly. We also showed that numerical precision for communication between compute nodes can be greatly reduced down to 16 or even 8 bit without introducing a large error. Reduced precision communication was not found to have a significant impact on either mean state, variability, geostrophy, or tendencies.

A *perfect model* is used in this study, such that any form of model or initial condition error is ignored and only the number format is changed between simulations. Solely discretization errors are estimated by lowering the spatial resolution by a factor of 2. Although this is essential here to analyze the impact of rounding errors isolated from other errors, it is in general not a realistic configuration for weather or climate models. More complex models include many other sources of forecast error, such that the contribution of rounding errors from 16‐bit arithmetic would likely be dwarfed by model, discretization, or initial condition errors.

Only the most common discretization method for fluid dynamics was used in this study: finite differences with an explicit time stepping scheme. But various other discretization methods exist, such as finite element or volume, spectral methods, and implicit time stepping. These methods come with different algorithms and associated precision requirements. Consequently, some might be less tolerant to rounding errors than the method used in this study.

There is currently no hardware available for posit arithmetic that we could have used for performance testing and it is seems impossible to make credible estimates whether such hardware would be faster or slower when compared to hardware‐optimized Float16 arithmetic, as this does not only depend on theoretical considerations but also on investments into chip design. We therefore cannot draw any conclusion about the performance of posit arithmetic operations in comparison to Float16 or the other formats.

Until progress is made on hardware implementations for posits, the results here suggest that also 16‐bit float arithmetic can successfully be used for parts of complex weather and climate models with the potential for acceleration on GPU and TPU. It is therefore recommended to adapt a type‐flexible programming paradigm, ideally in a language that supports portability, with algorithms written to reduce the dynamic range of arithmetic results. Hardware progress on central, GPU or TPU, with various numbers formats supported, can subsequently be utilized to accelerate weather and climate simulations.

## Data Availability

Scripts and software to reproduce this study are available in Klöwer (2020).
